# Development of Cellular High-Protein Foods: Third-Generation Yellow Pea and Red Lentil Puffed Snacks

**DOI:** 10.3390/foods11010038

**Published:** 2021-12-24

**Authors:** Nasibeh Y. Sinaki, Mustafa Tugrul Masatcioglu, Jitendra Paliwal, Filiz Koksel

**Affiliations:** 1 Food and Human Nutritional Sciences Department, University of Manitoba, Winnipeg, MB R3T 2N2, Canada; Nasibeh.younessinaki@umanitoba.ca; 2 Food Engineering Department, Tayfur Sokmen Campus, Hatay Mustafa Kemal University, Antakya 31034, Turkey; tmasatci@gmail.com; 3 Department of Biosystems Engineering, University of Manitoba, Winnipeg, MB R3T 2N2, Canada; J.Paliwal@umanitoba.ca

**Keywords:** expanded cellular snacks, extrusion, pulses texture, protein denaturation, starch gelatinization

## Abstract

This study aimed to evaluate how extrusion cooking conditions and microwave heating play a role in enhancing physical and thermal properties of third-generation expanded cellular snacks made from yellow pea (YP) and red lentil (RL) flours for the first time. Increasing temperature and moisture content during extrusion resulted in darker, crunchier and crispier products with higher expansion index (EI). Microwave heating after extrusion led to an increase in cell size and porosity of YP and RL products when qualitatively compared to extrusion alone. Additionally, extrusion followed by microwave heating resulted in extensive damage to starch granular structure and complete denaturation of proteins. Using microwave heating, as a fast and inexpensive process, following partial cooking with extrusion was demonstrated to greatly improve the physical and thermal properties of YP and RL snacks. Microwave heating following mild extrusion, instead of severe extrusion cooking alone, can potentially benefit the development of high quality nutritionally-dense expanded cellular snacks made from pulse flours.

## 1. Introduction

Plant protein-rich foods, such as those from pulses (e.g., peas, lentils, etc.), offer environmentally less resource-intensive and more humane alternatives to animal protein-rich foods [1]. In addition, processing pulses into appealing snack foods can provide healthier food options, and facilitate increased consumption of foods rich in plant proteins, as recommended by several health organizations, e.g., Health Canada [2]. The global market value of plant-based snacks was US$34.69 billion in 2019 with an annual growth rate of up to 8.7% forecasted for the coming years, i.e., expected to worth over US$73 billion by 2028 [3]. In this regard, extrusion, a high temperature-short time process which has been traditionally used for production of starchy snack foods, can also be used for formulating snacks rich in plant proteins [4,5,6,7]. However, when compared to their starch-rich counterparts, protein enriched extrudates are generally unappealing, e.g., less expanded and harder [8,9,10]. For example, in whole cereal-based extrudates, Pastor-Cavada et al. [9] reported a decrease in expansion when relatively higher protein containing legume flours were added to the formulae. A decrease in expansion together with an increase in density and hardness was also reported when rice flour extrudates were supplemented with soybean flour [10]. Therefore, certain adaptations during the manufacturing of high protein extrudates may be required to ensure satisfactory physical quality at elevated protein concentrations. Accordingly, process modifications such as the use of physical blowing agent assisted extrusion [4] and microwave heating in combination with extrusion [11,12] have been introduced.

Microwave heating is a relatively fast and inexpensive heating process [11]. During microwave heating, product moisture is converted to superheated steam which creates high pressure zones within the product. With the temperature rise, as the food matrix transits from glassy to rubbery state, it yields under pressure and expands [12]. The rapid dielectric heating advantage of microwaves, especially when combined with mild extrusion, can be used to develop new food products with novel structures and desired palatability, i.e., third-generation snacks [12]. Third-generation snacks are relatively easier and cheaper to produce than directly expanded snacks and require less storage space than directly expanded snacks due to the higher density of the former [11].

The effects of processing conditions on physical quality of starchy third-generation snacks, e.g., corn starch-based snacks with low protein content (<0.10 kg per kg dry flour), have been well understood [12]. However, there is very limited information available for snacks with higher protein contents (>0.20 kg protein per kg dry flour). The objective of this study was to investigate the effects of extrusion temperature, feed moisture content and the use of microwave heating on physical properties (i.e., overall expansion, colour, microstructure and texture), starch crystallinity and thermal properties of expanded cellular foods made from relatively higher protein ingredients (i.e., yellow pea and red lentil flours). To the best of our knowledge, this is the first study to explore the physical and thermal characteristics of the third-generation products made from pulse flours alone.

## 2. Materials and Methods

### 2.1. Materials

Red lentil (*Lens culinaris*) and yellow pea (*Pisum Sativum*) flours were provided by Ingredion Inc. (Westchester, IL, USA). The particle size distributions of the flours, as received from the manufacturer, were maximum 5% on U.S.S. 30 mesh (595 micron) sieve and maximum 10% through U.S.S. 50 mesh (297 microns) sieve for red lentil flour (HOMECRAFT 5135), and maximum 10% through U.S.S. 50 mesh (297 microns) sieve for yellow pea flour (HOMECRAFT 1135).

### 2.2. Proximate Composition Analyses

AACC International standard methods were used to analyze moisture (44–19.01), protein (46–30), ash (08–01) and lipid (30–25) contents of flours [13]. Total dietary fiber and starch contents were determined using AOAC Approved Methods 991–43 and 996–11, respectively [14]. Carbohydrate content was calculated by subtracting protein, ash, fat, and moisture contents from 100%.

### 2.3. Extrusion Cooking Process

Red lentil (RL) and yellow pea (YP) flours were extruded using a co-rotating twin-screw extruder (MPF19, APV Baker Ltd., Peterborough, UK) with a die orifice diameter of 2.3 mm and a 25:1 screw length-to-diameter ratio as described in detail by Koksel and Masatcioglu [4]. The screw configuration starting from the feeder to the die end was as follows: 9D (D = 19 mm) feed screw, 2D 60° forward paddle, 1D 30° reverse paddle, 2D lead screw, 2D feed screw, 1D 30° reverse paddle, 1D feed screw, 1D 60° forward paddle, 4D feed screw, 1D 30° forward paddle, 1D lead screw. The extruder barrel had five separate temperature-controlled zones where each zone was heated by external electric band heaters and the temperature of each zone was measured using thermocouples. Two different temperature profiles in the five heating zones of the extruder barrel extending from the feeder towards the die were as follows: (1) 60/70/80/90/100 °C and (2) 60/80/100/115/125 °C. Two different feed moisture contents (0.20 and 0.24 kg water per kg dry flour) were used. These temperatures and moisture contents were selected based on preliminary experiments and represent mild extrusion conditions to allow partial extrusion cooking (i.e., low temperature and high feed moisture content). Accordingly, at these mild conditions that do not necessarily favour expansion, the effect of extrusion cooking on heat labile components and temperature sensitive reactions such as Maillard reactions may be curtailed. Extrusion runs were performed in duplicate, on separate days to account for day-to-day differences in production, for each die temperature and feed moisture content. Dry feed and water rates were calibrated at the beginning of each extrusion run, and process variables (e.g., torque, die pressure) were closely monitored during extrusion cooking to ensure the reproducibility of duplicate extrusion runs. Extrudates were collected as long strings after the extruder reached steady-state conditions. At each extrusion run, for each extrusion condition studied, approximately 1 kg of extrudates was collected. Extrudate strings were initially cooled down to ambient temperature for 1 h, then dried in an air oven (Thermo Fisher Scientific, Heratherm Oven-OGS100, Dreieich, Germany) overnight at 50 °C to reach a moisture content of <0.10 kg water per kg dry extrudate. Given the relatively low die temperatures and high moisture contents chosen, the products emerging from the extruder were not expanded and only partially cooked and are referred to as second-generation products throughout this study.

Extrusion process variables (torque and die pressure) were recorded during extrusion cooking (Table 1). Specific mechanical energy (SME) was calculated according to Koksel and Masatcioglu [4] with actual screw speed of 250 rpm, rated screw speed of 500 rpm, motor power rating of 2.2 kW, and constant feed rate of 3 kg h^−1^.

### 2.4. Microwave Heating Process

The dried second-generation products were cut into 2 cm long pieces and individually placed in the centre of the turntable of a commercial microwave oven (Panasonic NN-SD980S, 1200 W, Shanghai, China). Each extrudate piece was heated for 40 s, as optimized for maximum expansion during preliminary experiments following Lee et al. [15]. These extruded then microwave heated products are referred to as third-generation products throughout this study.

### 2.5. Physical Properties

Overall expansion of the second- and third-generation products was evaluated by measurements of radial expansion index (EI) and density following Koksel and Masatcioglu [4] and Ryu and Ng [16], respectively. For the EI, ten products were randomly selected, their diameter measured with a digital caliper (accuracy 0.01 mm) and divided by the extruder die orifice diameter, i.e., 2.3 mm. Then, the average of EI for these ten replicates was reported for each product. For density measurements, a canola seed displacement method was used. The average density of each product was reported as the average of 5 replications.

Only the third-generation products were tested for their colour and textural quality attributes, since second-generation products were deemed partially cooked during extrusion and were not expanded. Product colour was measured using a colour spectrophotometer (CM-3500d, Konica Minolta, Osaka, Japan) following Koksel and Masatcioglu [4]. Briefly, third-generation products produced at a specific process condition were cut into cylindrical extrudate pieces of 1 cm length and vertically placed into a glass measuring container (3 cm wide and 1 cm high). The colour parameters are defined as darkness/lightness ($L^{*}$), greenness/redness ($a^{*}$), and blueness/yellowness ($b^{*}$). A reference of $L_{\mathrm{ref}}^{*}=0$, $a_{\mathrm{ref}}^{*}=0$ and $b_{\mathrm{ref}}^{*}=0$ was used (Zero calibration box, CM-A124, Konica Minolta, Osaka, Japan) following Luo et al. [17]. The overall colour difference (ΔE) between the third-generation products and the reference was calculated using the following equation [17]:

(1) $$\Delta E={\left[{\left(L^{*}-L_{\mathrm{ref}}^{*}\right)}^{2}+{\left(a^{*}-a_{\mathrm{ref}}^{*}\right)}^{2}+{\left(b^{*}-b_{\mathrm{ref}}^{*}\right)}^{2}\right]}^{0.5}$$

Textural attributes including hardness, crispiness and crunchiness were obtained using a Texture Profile Analyzer (TA-XT-plus, Stable Micro Systems, Gudalming, UK) with a 5 kg load cell and a 1 mm thick Warner-Bratzler shear blade probe. Fifteen third-generation products were randomly grouped into three and tested following the method of Luo et al. [17]. Briefly, extrudates were cut into 4 cm long pieces, individually placed on the equipment platform with a 90° shear angle to the blade. Textural properties were determined using the built-in equipment software (Exponent version 6, Stable Micro Systems, Gudalming, UK). Hardness, crispiness and crunchiness results were extracted from the Force (N) vs. Time (s) graph as the peak force (N), the number of positive peaks (dimensionless) and the linear distance of the force vs. time plot (N s), respectively. These parameters were obtained and reported as the average of the three groups for each third-generation product.

Cross-sectional microstructure of second- and third-generation products was evaluated using a Scanning Electron Microscope (SEM) (Quanta FEG 650, FEI, Hillsboro, OR, USA) following Koksel and Masatcioglu [4]. Five mm thick slices were cut from the products and placed on aluminum stubs with silver paste. Samples were coated with Au-Pd alloy by a cold sputter coater (Denton Vacuum, Desk II, Moorestown, NJ, USA). A magnification of 250× and acceleration voltage of 10 kV were used.

### 2.6. Starch Crystallinity and Thermal Properties

X-ray diffraction patterns of the YP and RL flours and their second- and third-generation products were obtained on a Rigaku SmartLab X-ray diffractometer (Shibuya-ku, Tokyo, Japan) equipped with a monochromator. The monochromator selects the Kα radiation from a copper target generated under 40 kV and 30 mA. The analysis was performed using 0.1542 nm radiation wavelength. Diffractograms were scanned over the 2θ angles of 5–40°, with a scan speed of 1° min^−1^ and step width was 0.02°. The percentage of starch relative crystallinity (RC) was calculated for each diffractogram by the following equation:

(2) $$\mathrm{RC}\left(\%\right)=\left(\frac{A_{c}}{A_{c}+A_{a}}\right)\times 100$$

where A_c_ is the area under the diffractogram corresponding to the crystalline portion and A_a_ is the area corresponding to the amorphous portion.

Thermal properties of the YP and RL flours and their second- and third-generation products were analyzed using a differential scanning calorimeter (DSC, DSC 1 STAR^e^ System, Mettler Toledo, Columbus, OH, USA) according to Masatcioglu et al. [18] with slight modifications. Briefly, 3 ± 0.05 mg of ground (<212 µm) samples were weighed into aluminum DSC pans. After adding deionized water (12 µL), the pans were hermetically sealed and allowed to stand overnight (~16 h) at 4 °C. Each equilibrated sample was placed in a standard DSC cell with an empty pan as a reference and heated at a rate of 10 °C min^−1^ from room temperature to 120 °C. Onset temperature (T_o_), peak temperature (T_p_), enthalpy of starch gelatinization (ΔH Peak I) and enthalpy of protein denaturation (ΔH Peak II) were calculated using the STAR^e^ Evaluation Software. Results were reported as means of duplicate analyses.

### 2.7. Statistical Analysis

Results were evaluated using Pearson correlations and one-way analysis of variance (ANOVA) using Minitab Statistical Software (Version 17, Minitab Corp., State College, PA, USA). Tukey pairwise comparison test was used to compare the means of product properties at 95% confidence intervals (*p* ≤ 0.05).

## 3. Results and Discussion

The effects of extruding RL and YP flours at two extrusion temperature profiles and moisture contents on torque, die pressure and SME are presented in Table 1. For both RL and YP flours, increasing the extrusion temperature and moisture content significantly (*p* ≤ 0.05) decreased the torque, die pressure and SME; except for the torque and SME values for YP flour at 0.20 kg water per kg dry flour and the torque, die pressure and SME values for YP flour at 0.24 kg water per kg dry flour where the decrease with temperature was not statistically significant. Similar results have also been reported by Onwulata et al. [19] where an increase in moisture content resulted in a decrease in torque, die pressure and SME values. An increase in torque values with a decrease in MC was evident for both flour types (r = −0.893 for RL and −0.850 for YP flours). Similarly, an inverse relationship between die temperature and SME values was also demonstrated by the high correlation coefficients, for both flour types (Supplementary Tables S1 and S2). The SME values varied from 167 to 297 Wh kg^−1^, in line with the literature for extrusion studies in similar temperature and moisture content range [20].

### 3.1. Proximate Composition

Table 2 shows the proximate composition of the raw red lentil (RL) and yellow pea (YP) flours. Based on these results, YP and RL flours had comparable starch contents. However, RL flour had approximately 3.2% higher protein and 2.3% lower dietary fiber content compared to YP flour. Similar results on starch and protein content of yellow pea and lentil flour have been reported previously by Li and Ganjyal [21]. Compared to wheat flour, which is commonly used in production of third-generation products, both YP and RL flours have higher protein and lower starch contents, i.e., protein content of ~9–10% and starch content of 60% in wheat flour [22].

### 3.2. Radial Expansion Index (EI) and Product Density

**Figure 1:**
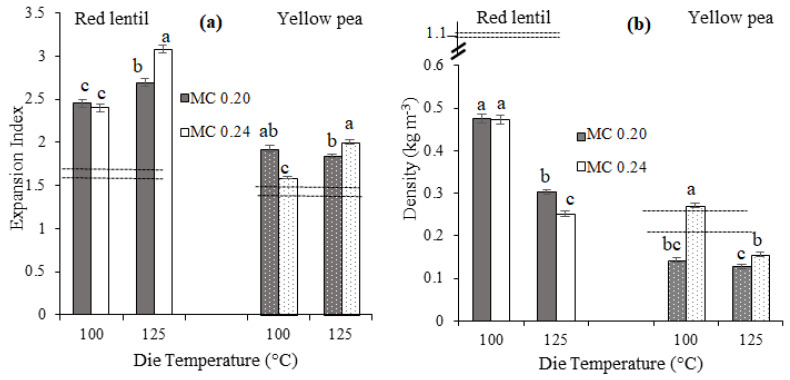
Expansion index (**a**) and density (**b**) of third-generation red lentil and yellow pea products with moisture contents of 0.20 (MC 0.20) and 0.24 (MC 0.24) kg water per kg dry flour. Dashed lines refer to the minimum and maximum expansion index (**a**) and density (**b**) of second-generation products. Error bars represent ± standard error. For each flour type, expansion index and density values designated with different letters are significantly different (*p* ≤ 0.05).

The effects of two main extrusion cooking factors, i.e., die temperature (DT) and feed moisture content (MC) on third-generation product expansion are presented in Figure 1. The minimum and maximum EI values for second-generation products were 1.45 ± 0.01 (mean ± standard error) and 1.73 ± 0.01, demonstrated as dashed lines in Figure 1a, while for the third-generation products these values were 1.59 ± 0.01 and 3.08 ± 0.05. As expected, with microwave heating, the third-generation products had greater expansion compared to the second-generation products that were extrusion cooked only. In the literature, EI values of extrusion cooked products at similar DT (i.e., 120 °C) have been reported as high as 2.2 for 60% chickpea and 40% sorghum blends (protein content of 16.9% d.b.) [1], which is higher than those obtained in our study. Not surprisingly, when raw materials with lower protein content are extruded, the EI values of extrusion cooked products display larger values. For example, incorporating 15% and 30% navy bean flour to corn starch to achieve total protein contents of 4.8% and 8.4% d.b. reduced the EI values from 2.5 to 2.1 and 1.8, respectively [23]. The EI values of protein enriched third-generation products were also somewhat lower than those of conventional starch-rich products. For example, a starchy third-generation product made from blue corn and corn starch (protein content of 5.8% d.b.) was reported to have an EI of 4.8 [24], while the EI of a third-generation product made from a higher protein content blend of potato starch, corn protein and soybean meal (protein content of 11.5% d.b.) was reported as 3.3 at an extrusion die temperature of 85 °C [25].

An increase in extrusion DT resulted in a significant (*p* ≤ 0.05) increase in EI values of third-generation products, except for YP with MC of 0.20 kg water per kg dry flour, where no significant change in EI was observed (Figure 1a). An increase in starch gelatinization due to increasing DT [18,26] is possibly responsible for the observed higher expansion [12]. This is in line with the findings from thermal analysis of third-generation products (see Section 3.6 Starch crystallinity and thermal properties). In the case of YP extrudates with MC 0.20 kg water per kg dry flour, it is longitudinal expansion that was likely more dominant over radial expansion so that no significant change in EI due to increasing DT was observed. For both RL and YP flours, at 100 °C, EI stayed the same or decreased with increasing MC, while an opposite trend was observed at 125 °C (Figure 1a). The decrease in EI with increasing MC at 100 °C is possibly due to a decrease in melt’s resistance to flow (i.e., lower apparent viscosity) in the extruder barrel because of water’s plasticizing effect which is also reflected as lower SME values (Table 1). Accordingly, a higher MC may lead to structural collapse as the extrudate leaves the die as well as a decrease in overall extrudate expansion [12,20]. Product expansion was also a function of flour type (i.e., RL vs. YP). Based on Figure 1a, third-generation RL products expanded to a greater extent compared to YP products. This greater expansion observed in RL products can be attributed to the slightly lower total dietary fiber content of RL flour compared to that of YP flour, since higher total dietary fiber has been shown to reduce overall expansion [12,27].

In Figure 1b, density of the second- and third-generation RL and YP products as a function of DT and MC are presented. Density values for the second-generation products ranged from 0.21 ± 0.01 to 1.1 ± 0.04 kg m^−3^ while the density for third-generation products ranged from 0.13 ± 0.01 to 0.48 ± 0.01 kg m^−3^ (Figure 1b). As expected, for all products, density was equal or higher for the second-generation products compared to the third-generation products, in line with previously reported results [28].

Density of third-generation products significantly decreased (*p* ≤ 0.05) with increasing DT (Figure 1b), reflecting changes in extrudate expansion both in the radial and longitudinal directions. The only exception to this trend was YP with MC of 0.20 kg water per kg dry flour where no significant change in density was observed. A direct relationship between EI and density of YP extrudates produced at 125 °C was observed, i.e., both EI and density increased with increasing MC. This direct relationship, contrary to the commonly reported inverse relationship between EI and density might be due to the more dominant role of longitudinal expansion in extrudate density. Depending on the extent to which the longitudinal expansion contributes to the final volume of extrudates, the inverse relationship between EI and density might no longer exist. With an increase in moisture content, no significant change in the density of third-generation RL products was observed at 100 °C, while a decrease in density was observed at 125 °C (Figure 1b), indicating that the extent to which MC affects product density increased at higher temperature. This decrease in density is likely due to the higher temperature enhancing MC’s effect on starch gelatinization/melting, and subsequently affecting overall expansion [20]. For third-generation YP products, a different trend was observed: the density increased with increasing MC from 0.20 to 0.24 kg water per kg dry flour, at both DTs. Increasing MC may reduce the apparent viscosity of melt in the extruder barrel resulting in the increase in density and decrease in SME (Table 1). Moreover, although increasing MC has been shown to generally increase product density in starchy extrudates [29], non-starch components in pulses such as fiber and proteins interact with how moisture is absorbed and distributed during processing, affecting expansion [24]. The different trend in the effect of MC on density of third-generation RL and YP products is possibly related to the difference in the total dietary fiber contents of RL and YP flours. Fiber molecules can bind moisture, reducing the availability of water for other components (e.g., starch), and thus may reduce expansion and increase density [12,27]. Consequently, increasing moisture content in YP products with higher fiber content compared to RL products led to an increase in their density.

### 3.3. Colour

Colour is an important quality parameter of snack foods that greatly affects the visual appeal of the final product [30]. The impact of the DT and MC on the third-generation product colour is presented in Table 3. RL products produced at 100 °C were brighter (higher $L^{*}$ value), less red (lower $a^{*}$ value), less yellow (lower $b^{*}$ value) and had higher ΔE values when compared to their counterparts produced at 125 °C, at the same MC. In general, for third-generation products made from both flours, die temperature and $a^{*}$ values were positively correlated (r = 0.903 for RL and 0.696 for YP flours) meaning that as the die temperature increased products became more red (Supplementary Tables S1 and S2). It has been shown that Maillard reactions increase with an increase in temperature, leading to lower $L^{*}$ and higher $a^{*}$ and $b^{*}$ values (i.e., darker, more red and more yellow products) [31]. Accordingly, the decrease in the ΔE values of RL products with increasing DT is possibly a result of the higher rate of Maillard reactions in the extrudates at 125 °C. Similarly, Nam [30] reported that the $L^{*}$ values of pea starch extrudates decreased with an increase in extrusion temperature while the $a^{*}$ values showed the opposite trend.

Among all the studied products, third-generation RL products produced at MC of 0.24 kg water per kg dry flour and temperature of 100 °C showed the highest $L^{*}$ value, i.e., the lightest colour. These products also had the lowest $a^{*}$ value (indicating the lowest red hue) and the highest ΔE value (suggesting the colour being farthest to the black reference colour). This possibly indicates a lower rate of Maillard reactions at these processing conditions, since it has been shown that Maillard reactions are favoured at high temperature and low moisture content conditions [31]. The lowest ΔE was found for RL products with 0.20 kg water per kg dry flour produced at 125 °C, suggesting that these products were the closest to the reference colour (i.e., black). Total colour difference (ΔE) varied from 84.9 to 88.6 for all third-generation products studied (Table 3).

### 3.4. Cross-Sectional Microstructure

**Figure 2:**
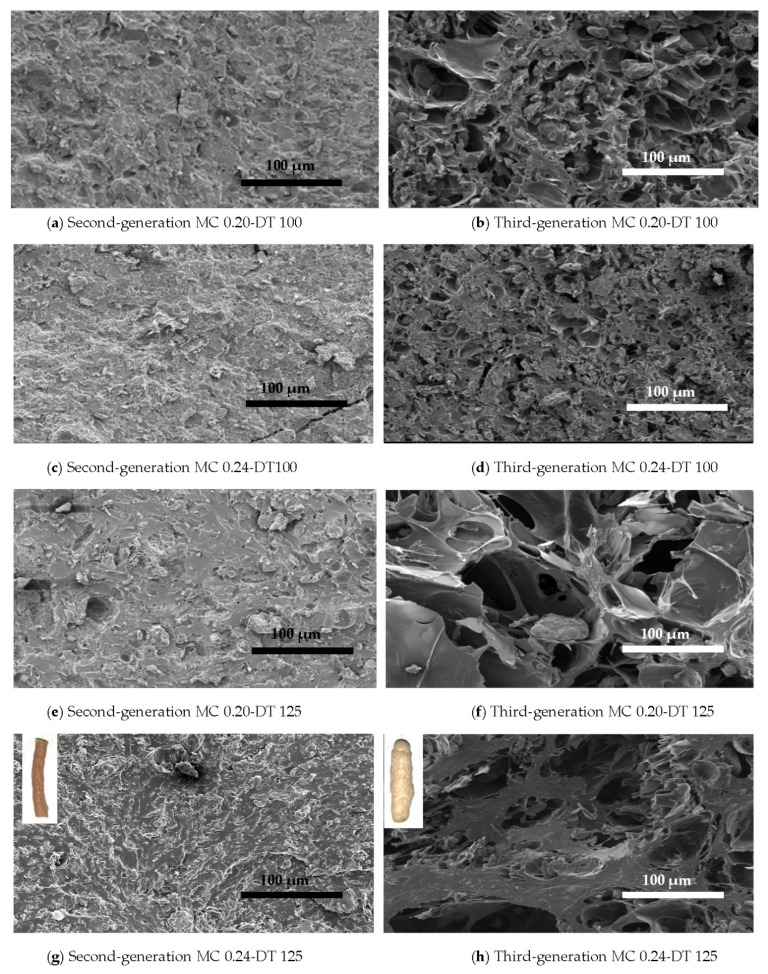
Scanning electron micrographs of second-generation (first column) and third-generation (second column) red lentil products with feed moisture contents of 0.20 and 0.24 kg water per kg dry flour (MC 0.20 and MC 0.24, respectively) at 100 °C and 125 °C die temperature (DT 100 and DT 125, respectively). A 250× magnification was used for all images with a scale bar of 100 µm shown. Digital images in (**g**,**h**) show red lentil second- and third-generation products, respectively, produced with MC of 0.24 at 125 °C.

The cross-sectional microstructure images of second- and third-generation RL products are presented in Figure 2 (as representative of both YP and RL products). Considering that both second- and the third-generation products were magnified to the same degree, the second-generation products had relatively smaller air cells while the third-generation ones showed a more porous cellular structure with relatively larger air cells. The digital images of second- and third-generation products at 0.24 kg water per kg dry flour MC and 125 °C (overlayed onto the top right corners of the Figure 2g,h, respectively) also support the increase in expansion with microwave heating, and indirectly the larger air cells in third-generation products. Similar trends in microstructure have been reported by Lee et al. [15] who studied extruded and microwaved starch pellets. These changes in microstructure of second- and third-generation products are due to the rapid evaporation of water and creation of local regions of high pressure superheated steam by microwave heating that create a microstructure with larger air cells [12].

### 3.5. Texture of Third-Generation Products

**Figure 3:**
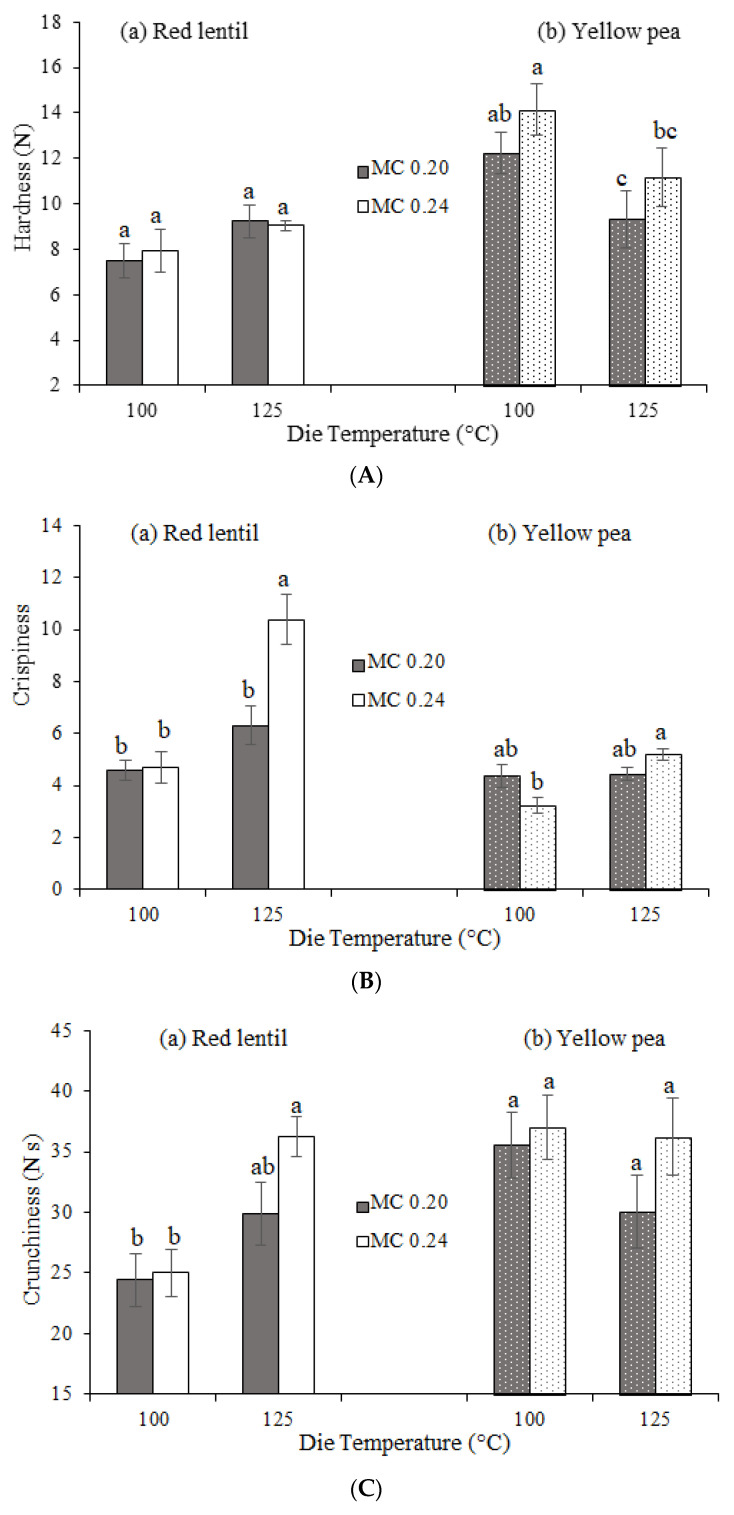
(**A**) Hardness, (**B**) crispiness, and (**C**) crunchiness values of third-generation (a) red lentil and (b) yellow pea products with moisture contents of 0.20 and 0.24 kg water per kg dry flour (MC 0.20 and MC 0.24, respectively). Error bars represent ± standard error. For each flour type, texture parameters designated with different letters are significantly different (*p* ≤ 0.05).

The effects of DT and MC on third-generation product textural attributes, i.e., hardness, crispiness and crunchiness, are presented in Figure 3. Since the second-generation products were only semi-finished (i.e., partially cooked), their textural properties were not measured. For RL products, hardness values were not significantly affected by DT or MC. For YP products, hardness significantly decreased with increasing DT (*p* ≤ 0.05, Figure 3). This reduction in hardness of YP products might be attributable to an increase in starch gelatinization of the YP products with increasing DT (Table 4). It has been shown that an increase in starch gelatinization can increase expansion and decrease the hardness values of extrudates [12]. Another reason for the lower hardness values observed for YP products at higher DT is possibly a result of the increase in vapor pressure of water with increasing temperature that promotes cell size growth [29], resulting in overall greater expansion and thus lowering hardness. Similarly, Aguilar-Palazuelos et al. [25] also showed that an increase in extrusion temperature resulted in reduced hardness values in microwave expanded pellets made from blends of potato starch, corn protein and soybean meal. However, an increase in starch gelatinization or water vapor pressure alone does not explain the differences observed for the third-generation RL and YP products. The different trend of the effect of DT on hardness of these products is possibly related to the difference in the non-starchy ingredients such as total dietary fiber contents of RL and YP flours (in this study, YP flour fiber content is greater than that of RL flour). It has been shown that with an increase in extrusion temperature some of the insoluble fibers may become soluble decreasing the hardness of extrudates [32,33]. Consequently, increasing the temperature for YP products with higher fiber content might have led to the transformation of insoluble to soluble fibers, and thus decreased their hardness.

Crispiness generally increased with increasing temperature for both RL and YP products. However, this increase was only statistically significant (*p* ≤ 0.05) for third-generation products containing higher moisture, i.e., 0.24 kg water per kg dry flour moisture content (Figure 3). No significant difference was found in the crunchiness of products studied, except for RL products produced at 125 °C and 0.24 kg water per kg dry flour moisture content for which higher crunchiness was observed when compared to the rest of the RL products studied (Figure 3). The effect of increasing temperature on the increase in crispiness is again probably due to the promotion of cell size growth arising from an increase in the vapor pressure of water with increasing temperature [29]. With an increase in expansion and a reduction in density, products become more porous and the number of breaking points on cell walls increase on average, causing a higher level of perceived crispiness [29]. The increase in expansion and cell size growth with increasing temperature is in line with EI, density and microstructure results of third-generation products.

### 3.6. Starch Crystallinity and Thermal Properties

The X-ray diffraction patterns of RL and YP flours and their products are presented in Supplementary Material (Supplementary Figure S1). While RL flour showed diffraction peaks at 15.0, 17.6 and 22.8° (2θ), the respective 2θ values for YP flour were 15.0, 17.0 and 23.4°. Hence, both flours showed C-type diffraction patterns characteristic of pulse starches. The relative crystallinity (RC) calculated based on diffraction intensity was lower for RL flour (14.9%) than that of YP flour (17.5%). These values are lower than the RC values reported by Zhou et al. [34] for C-type pulse starches ranging between 27.1–33.5%, possibly due to the utilization of flour samples in X-ray diffraction analysis in the present study instead of their isolated starches. For second-generation products, the diffraction peaks completely disappeared, suggesting that the starch in both the RL and YP extrudates lost their crystallinity under the studied extrusion conditions, due to the mechanical and thermal energy input during extrusion cooking. Further treatment by microwave heating of products (i.e., third-generation products) did not substantially affect the shape of the diffractograms.

The DSC thermograms of raw RL and YP flours showed two endothermic peaks (Table 4 and Supplementary Figure S2), the first one (Peak I) corresponding to starch gelatinization and the second one (Peak II) to protein denaturation [35]. For starch gelatinization (Peak I), the peak temperature (T_p_) value of the second-generation RL products produced at the MC of 0.20 and 0.24 kg water per kg dry flour and DT of 100 and 125 °C were substantially lower compared to the T_p_ of raw RL flour. Moreover, change in enthalpy (ΔH) values of these second-generation products were drastically lower compared to that of RL flour, indicating that starch was gelatinized to a large extent due to the mechanical and thermal energy input during extrusion cooking. The cross-sectional images (Figure 2) of the second-generation products show that starch granules considerably melted and plasticized, specifically at the higher DT, i.e., 125 °C, and higher MC, i.e., 0.24 kg water per kg dry flour, during extrusion. The T_p_ and ΔH properties of the second-generation YP products showed a similar trend with their second-generation RL counterpart compared to the raw material. This trend is also supported by the loss of crystallinity peaks in the X-ray diffraction patterns of these extrudates (Supplementary Figure S1).

For the starch gelatinization (Peak I), the enthalpy (ΔH) values of the third-generation YP products were much lower compared to those of the second-generation ones (Table 4). This decrease in ΔH was more evident in products extruded both at higher DT and higher MC, i.e., 125 °C and 0.24 kg water per kg dry flour. In other words, increasing DT and MC resulted in an increase in the degree of gelatinization, and thus a decrease in the gelatinization enthalpy and gelatinization temperature range (between T_o_ and T_p_), in line with the findings of Jafari et al. [20]. Although no crystallinity was observed in the second- and third-generation products (Supplementary Figure S1), gelatinization peaks, albeit small, were observed in the DSC thermograms (Table 4, Peak I) indicating residual granular structure after extrusion and microwave processing. Hence, a comparison of the results of X-ray diffractometry and DSC revealed that DSC is much more sensitive in characterizing starch’s thermal transitions. For both RL and YP third-generation products, the enthalpy (ΔH) values for starch gelatinization were positively correlated with extrusion variables, i.e., torque (r = 0.912 for RL and 0.879 for YP products), die pressure (r = 0.910 for RL and 0.886 for YP products) and SME (r = 0.881 for RL and 0.826 for YP products). These results (Supplementary Table S1) indicate that as the torque, die pressure and/or SME increased a higher level of starch gelatinization occurred during extrusion.

For protein denaturation (Peak II), ΔH values of the second-generation RL and YP products were lower compared to that of their raw flours, indicating that protein was denatured to some extent during extrusion. The decrease in ΔH with extrusion was notably greater at the higher DT. For second-generation RL products, protein denaturation was more severe when compared to YP products, i.e., Peak II was only observed at the lower DT and lower MC. It should be noted that Peak II was only observed for some and not detected for all second-generation products, meaning that complete protein denaturation was achieved when the peak is not detected. Ai et al. [35] reported that extrusion cooking caused complete starch gelatinization and protein denaturation of all common bean powders they studied at slightly higher extrusion temperatures (120 and 140 °C) than the present study. For the protein denaturation (Peak II), the third-generation YP and RL products did not exhibit any thermal transition in their DSC thermograms. Therefore, their proteins can be considered completely denatured after microwave heating.

## 4. Conclusions

The effects of microwave heating on expansion index, density, colour, microstructure, texture, starch crystallinity and gelatinization, and protein denaturation of YP and RL extrudates were investigated. The results showed that microwave heating enhanced the physical and thermal properties of the final products, e.g., overall increase in expansion and decrease in density, further starch damage accompanied by protein denaturation. The outcomes of this study can be used as a basis for the production of third-generation pulse-based snacks with desired texture by the food industry even at low extrusion temperature and high moisture contents that are typically used in the manufacture of starchy snack foods.

## Figures and Tables

**Table 1:** Effects of feed moisture content and die temperature (DT) on torque, die pressure and specific mechanical energy (SME) values of extrudates made from red lentil (RL) and yellow pea (YP) flours. For each flour type, results in each column are significantly different if designated with different lowercase letters (*p* ≤ 0.05).

Flour	Moisture Content	DT	Torque	Die Pressure	SME
	(kg water per kg dry flour)	(°C)	(%)	(kPa)	(Wh kg^−1^)
RL	0.20	100	34 a	7100 a	297 a
		125	29 b	5350 c	257 b
	0.24	100	25 c	5900 b	220 c
		125	21 d	4600 d	187 d
YP	0.20	100	25 a	8150 a	218 a
		125	22 ab	6600 b	196 ab
	0.24	100	21 bc	5950 bc	182 bc
		125	19 c	5100 c	167 c

**Table 2:** Proximate composition (mean ± standard error) of red lentil and yellow pea flours.

Composition (g per 100 g dry flour)	Red Lentil	Yellow Pea
Protein	25.11 ± 0.37	21.89 ± 0.43
Ash	3.37 ± 0.01	3.50 ± 0.05
Lipid	1.29 ± 0.03	0.80 ± 0.02
Total carbohydrates	70.23	73.81
Total dietary fiber	11.21 ± 0.73	13.54 ± 0.84
Starch	52.42 ± 0.60	52.20 ± 0.51

**Table 3:** Colour parameters (mean ± standard error) of third-generation red lentil (RL) and yellow pea (YP) products as affected by feed moisture content and die temperature (DT). For each flour type, results in each column are significantly different if designated with different lowercase letters (*p* ≤ 0.05).

Flour	Moisture Content (kg water per kg dry flour)	DT (°C)	Colour Parameters			
			$L^{*}$	$a^{*}$	$b^{*}$	ΔE
RL	0.20	100	85.1 ± 0.1 a	0.8 ± 0.0 c	23.8 ± 0.4 b	88.4 ± 0.0 ab
		125	80.9 ± 0.6 c	2.7 ± 0.1 a	25.4 ± 0.2 a	84.9 ± 0.6 c
	0.24	100	85.7 ± 0.1 a	0.6 ± 0.1 c	22.6 ± 0.2 c	88.6 ± 0.0 a
		125	83.6 ± 0.3 b	1.8 ± 0.1 b	24.2 ± 0.3 b	87.1 ± 0.3 b
YP	0.20	100	84.5 ± 0.1 a	2.6 ± 0.0 c	25.5 ± 0.0 b	88.3 ± 0.1 a
		125	84.2 ± 0.2 ab	2.8 ± 0.1 b	26.03 ± 0.3 b	88.2 ± 0.1 ab
	0.24	100	83.2 ± 0.5 bc	2.7 ± 0.1 bc	26.5 ± 0.4 ab	87.4 ± 0.3 bc
		125	82.7 ± 0.3 c	3.4 ± 0.1 a	27.5 ± 0.3 a	87.3 ± 0.2 c

**Table 4:** Thermal properties of red lentil (RL) and yellow pea (YP) flours and their products as a function of die temperature (DT) and moisture content.

Sample		DT	Moisture Content	Peak I			Peak II		
		(°C)	(kg water per kg dry flour)	T_o_ (°C)	T_p_ (°C)	ΔH (J/g)	T_o_ (°C)	T_p_ (°C)	ΔH (J/g)
RL	Flour (raw)			59.3 ± 0.1	66.2 ± 0.0	4.20 ± 0.3	79.9 ± 0.4	85.5 ± 0.0	0.52 ± 0.0
	Second-generation	100	0.20	44.2 ± 0.4	55.3 ± 0.0	1.44 ± 0.0	84.5 ± 0.1	91.8 ± 0.0	0.48 ± 0.0
			0.24	47.2 ± 2.8	54.4 ± 2.7	1.53 ± 0.0	nd	nd	nd
		125	0.20	46.5 ± 1.1	56.8 ± 0.8	1.33 ± 0.0	nd	nd	nd
			0.24	47.7 ± 0.7	57.2 ± 0.1	1.21 ± 0.1	nd	nd	nd
	Third-generation	100	0.20	49.9 ± 0.3	56.4 ± 1.4	0.46 ± 0.0	nd	nd	nd
			0.24	51.4 ± 0.0	56.0 ± 0.0	0.26 ± 0.1	nd	nd	nd
		125	0.20	51.5 ± 0.3	57.7 ± 0.4	0.27 ± 0.0	nd	nd	nd
YP	Flour (raw)			62.3 ± 0.1	69.4 ± 0.0	4.57 ± 0.1	81.8 ± 0.2	86.2 ± 0.0	0.26 ± 0.0
	Second-generation	100	0.20	41.9 ± 0.7	55.9 ± 0.0	1.08 ± 0.1	74.4 ± 0.1	79.2 ± 0.0	0.23 ± 0.0
			0.24	45.6 ± 0.0	54.5 ± 0.0	0.93 ± 0.0	75.0 ± 0.0	78.7 ± 0.0	0.13 ± 0.0
		125	0.20	46.9 ± 0.9	56.7 ± 1.1	0.93 ± 0.0	86.4 ± 0.8	91.0 ± 1.2	0.04 ± 0.0
			0.24	46.4 ± 0.8	56.1 ± 3.2	0.84 ± 0.0	nd	nd	nd
	Third-generation	100	0.20	53.9 ± 4.8	58.4 ± 0.4	0.35 ± 0.0	nd	nd	nd
			0.24	54.3 ± 0.5	58.4 ± 2.2	0.21 ± 0.0	nd	nd	nd
		125	0.20	53.2 ± 1.3	59.2 ± 0.7	0.24 ± 0.0	nd	nd	nd
			0.24	54.3 ± 0.3	58.1 ± 0.9	0.14 ± 0.0	nd	nd	nd

T_o_: Onset temperature; T_p_: Peak temperature; ΔH: Enthalpies of starch gelatinization and protein denaturation for Peaks I and II, respectively; nd: not detected.

## Data Availability

The data that support the findings of this study are available on request from the corresponding author.

## Supplementary Materials

The following are available online at https://www.mdpi.com/article/10.3390/foods11010038/s1, Figure S1: X-ray diffraction patterns of second-generation (a and b) and third-generation (c and d) yellow pea (YP) and red lentil (RL) products with feed moisture contents of 0.20 and 0.24 kg water per kg dry flour (MC0.20 and MC0.24, respectively) at 100 °C and 125 °C die temperature (DT100 and DT125, respectively); Figure S2: DSC thermograms of red lentil (RL) and yellow pea (YP) flours, second-generation and third-generation red lentil and yellow pea products with feed moisture content of 0.20 kg water per kg dry flour (MC0.20) at 100 °C die temperature (DT100). Table S1 Pearson correlation coefficients between extrusion variables and extrudate properties in red lentil (RL) third-generation products. Table S2 Pearson correlation coefficients between extrusion variables and extrudate properties in yellow pea (YP) third-generation products.

## References

[1] Wang S., Nosworthy M.G., House J.D., Ai Y., Hood-Niefer S., Nickerson M.T. (2019). Effect of barrel temperature and feed moisture on the physical properties of chickpea–sorghum and chickpea–maize extrudates, and the functionality and nutritional value of their resultant flours—Part II. Cereal Chem..

[2] Health Canada (2019). Healthy Eating Recommendations. https://food-guide.canada.ca/en/healthy-eating-recommendations/.

[3] Plant-Based Snacks Market, Analysis and Review Plant-Based Snacks Market by Nature—Organic and Conventional for 2019–2028. https://www.futuremarketinsights.com/reports/plant-based-snacks-market.

[4] Koksel F., Masatcioglu M.T. (2018). Physical properties of puffed yellow pea snacks produced by nitrogen gas assisted extrusion cooking. LWT Food Sci. Technol..

[5] Pasqualone A., Costantini M., Coldea T.E., Summo C. (2020). Use of legumes in extrusion cooking: A review. Foods.

[6] Pasqualone A., Costantini M., Labarbuta R., Summo C. (2021). Production of extruded-cooked lentil flours at industrial level: Effect of processing conditions on starch gelatinization, dough rheological properties and techno-functional parameters. LWT Food Sci. Technol..

[7] Proserpio C., Bresciani A., Marti A., Pagliarini E. (2020). Legume flour or bran: Sustainable, fiber-rich ingredients for extruded snacks. Foods.

[8] Chaiyakul S., Jangchud K., Jangchud A., Wuttijumnong P., Winger R. (2009). Effect of extrusion conditions on physical and chemical properties of high protein glutinous rice-based snack. LWT Food Sci. Technol..

[9] Pastor-Cavada E., Drago S.R., González R.J., Juan R., Pastor J.E., Alaiz M., Vioque J. (2011). Effects of the addition of wild legumes (*Lathyrus annuus* and *Lathyrus clymenum*) on the physical and nutritional properties of extruded products based on whole corn and brown rice. Food Chem..

[10] Suksomboon A., Limroongreungrat K., Sangnark A., Thititumjariya K., Noomhorm A. (2011). Effect of extrusion conditions on the physicochemical properties of a snack made from purple rice (*Hom Nil*) and soybean flour blend. Int. J. Food Sci. Technol..

[11] Aguilar-Palazuelos E., Zazueta-Morales J., Martínez-Bustos F. (2006). Preparation of high-quality protein-based extruded pellets expanded by microwave oven. Cereal Chem..

[12] Moraru C.I., Kokini J.L. (2003). Nucleation and expansion during extrusion and microwave heating of cereal foods. Compr. Rev. Food Sci. Food Saf..

[13] AACC International (2000). Approved Methods of American Association of Cereal Chemists (AACC) Methods 44-01, 08-01, 30-25, and 46-30.

[14] Association of Official Analytical Chemists (2000). Methods 991.43 and 996.11. Official Methods of Analysis.

[15] Lee E.Y., Lim K., Lim J.K., Lim S.L. (2000). Effects of gelatinization and moisture content of extruded starch pellets on morphology and physical properties of microwave-expanded products. Cereal Chem..

[16] Ryu G.H., Ng P.K.W. (2001). Effects of selected process parameters on expansion and mechanical properties of wheat flour and whole cornmeal extrudates. Starch Stärke.

[17] Luo S., Chan E., Masatcioglu M.T., Erkinbaev C., Paliwal J., Koksel F. (2020). Effects of extrusion conditions and nitrogen injection on physical, mechanical, and microstructural properties of red lentil puffed snacks. Food Bioprod. Process..

[18] Masatcioglu T., Yalcin E., Hwan P.J., Ryu G.H., Celik S., Koksel H. (2014). Hull-less barley flour supplemented corn extrudates produced by conventional extrusion and CO_2_ injection process. Innov. Food Sci. Emerg. Technol..

[19] Onwulata C.I., Smith P.W., Konstance R.P., Holsinger V.H. (2001). Incorporation of whey products in extruded corn, potato or rice snacks. Food Res. Int..

[20] Jafari M., Koocheki A., Milani E. (2017). Effect of extrusion cooking on chemical structure, morphology, crystallinity and thermal properties of sorghum flour extrudates. J. Cereal Sci..

[21] Li C., Ganjyal G.M. (2017). Chemical composition, pasting, and thermal properties of 22 different varieties of peas and lentils. Cereal Chem..

[22] Sramkova Z., Gregova E., Sturdik E. (2009). Chemical composition and nutritional quality of wheat grain. Acta Chim. Slovaca.

[23] Anton A.A., Fulcher R.G., Arntfield S.D. (2009). Physical and nutritional impact of fortification of corn starch-based extruded snacks with common bean (*Phaseolus vulgaris* L.) flour: Effects of bean addition and extrusion cooking. Food Chem..

[24] Camacho-Hernández I.L., Zazueta-Morales J.J., Gallegos-Infante J.A., Aguilar-Palazuelos E., Rocha-Guzmán N.E., Navarro-Cortez R.O., Jacobo-Valenzuela N., Gomez-Aldapa C.A. (2014). Effect of extrusion conditions on physicochemical characteristics and anthocyanin content of blue corn third-generation snacks. CyTA J. Food.

[25] Aguilar-Palazuelos E., Zazueta-Morales J.J., Harumi E.N., Martínez-Bustos F. (2012). Optimization of extrusion process for production of nutritious pellets. Food Sci. Technol..

[26] Chang Y.H., Ng P.K.W. (2011). Effects of extrusion process variables on quality properties of wheat-ginseng extrudates. Int. J. Food Prop..

[27] Sman R.G.M., Bows J.R. (2017). Critical factors in microwave expansion of starchy snacks. J. Food Eng..

[28] Panak Balentić J., Jozinović A., Ačkar Đ., Babić J., Miličević B., Benšić M., Jokić S., Šarić A., Šubarić D. (2019). Nutritionally improved third generation snacks produced by supercritical CO_2_ extrusion I. Physical and sensory properties. J. Food Process. Eng..

[29] Ding Q.B., Ainsworth P., Tucker G., Marson H. (2005). The effect of extrusion conditions on the physicochemical properties and sensory characteristics of rice-based expanded snacks. J. Food Eng..

[30] Nam S. (2002). Extrusion Technology for the Development of Barley Cereal Products and Bioactive Packaging Materials. Master’s Thesis.

[31] Ames J.M. (1990). Control of the Maillard reaction in food systems. Trends Food Sci. Technol..

[32] Ačkar Đ., Jozinović A., Babić J., Miličević B., Panak Balentić J., Šubarić D. (2018). Resolving the problem of poor expansion in corn extrudates enriched with food industry by-products. Innov. Food Sci. Emerg. Technol..

[33] Zhang M., Bai X., Zhang Z. (2011). Extrusion process improves the functionality of soluble dietary fiber in oat bran. J. Cereal Sci..

[34] Zhou Y., Hoover R., Liu Q. (2004). Relationship between α-amylase degradation and the structure and physicochemical properties of legume starches. Carbohydr. Polym..

[35] Ai Y., Cichy K.A., Harte J.B., Kelly J.D., Ng P.K.W. (2016). Effects of extrusion cooking on the chemical composition and functional properties of dry common bean powders. Food Chem..

