# Supplementary material for: Development of Cellular High-Protein Foods: Third-Generation Yellow Pea and Red Lentil Puffed Snacks
Source: Foods. 2021 Dec 24;11(1):38. doi: 10.3390/foods11010038 (PMC8750491; doi:10.3390/foods11010038)
Supplement: Supplementary file 1 [file foods-11-00038-s001.zip › foods-1491689-SI.pdf]

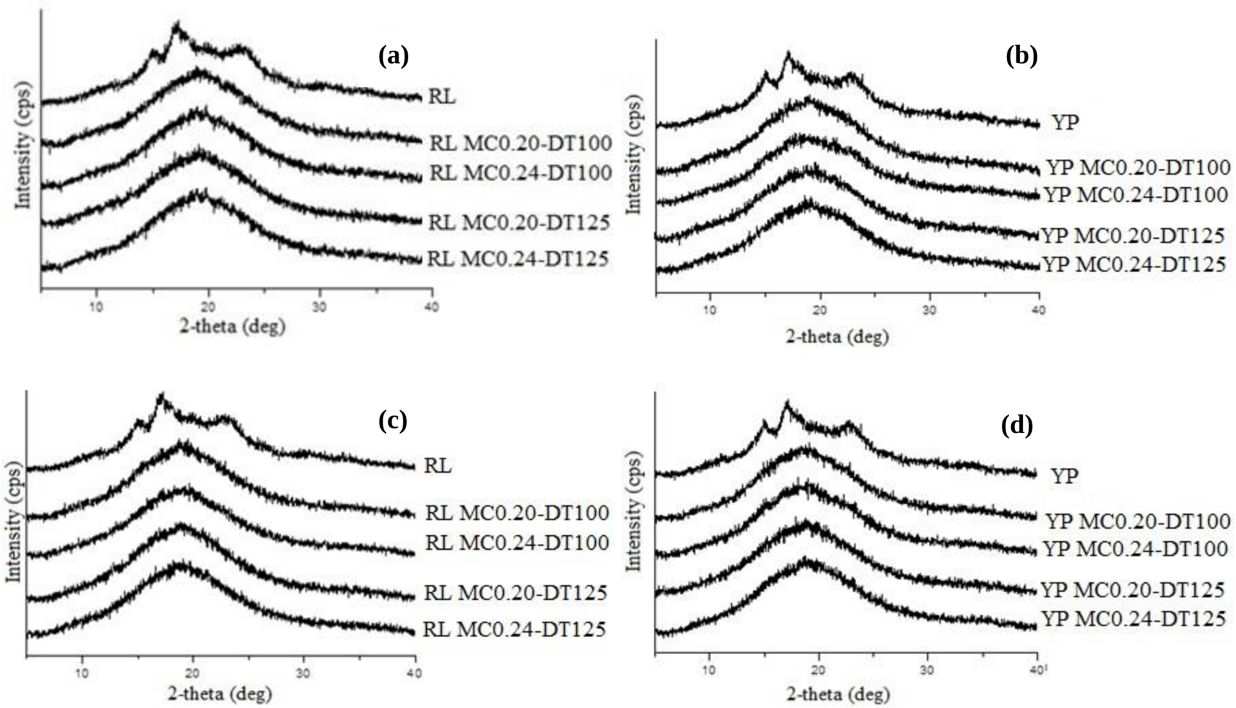

Figure S1. X-ray diffraction patterns of second-generation (a and b) and third-generation (c and d) yellow pea (YP) and red lentil (RL) products with feed moisture contents of 0.20 and 0.24 kg water per kg dry flour (MC0.20 and MC0.24, respectively) at 100 °C and 125 °C die temperature (DT100 and DT125, respectively).

Red Lentil | Yellow Pea

Flour (raw)

Second-generation MC0.20-DT100

Third-generation MC0.20-DT100

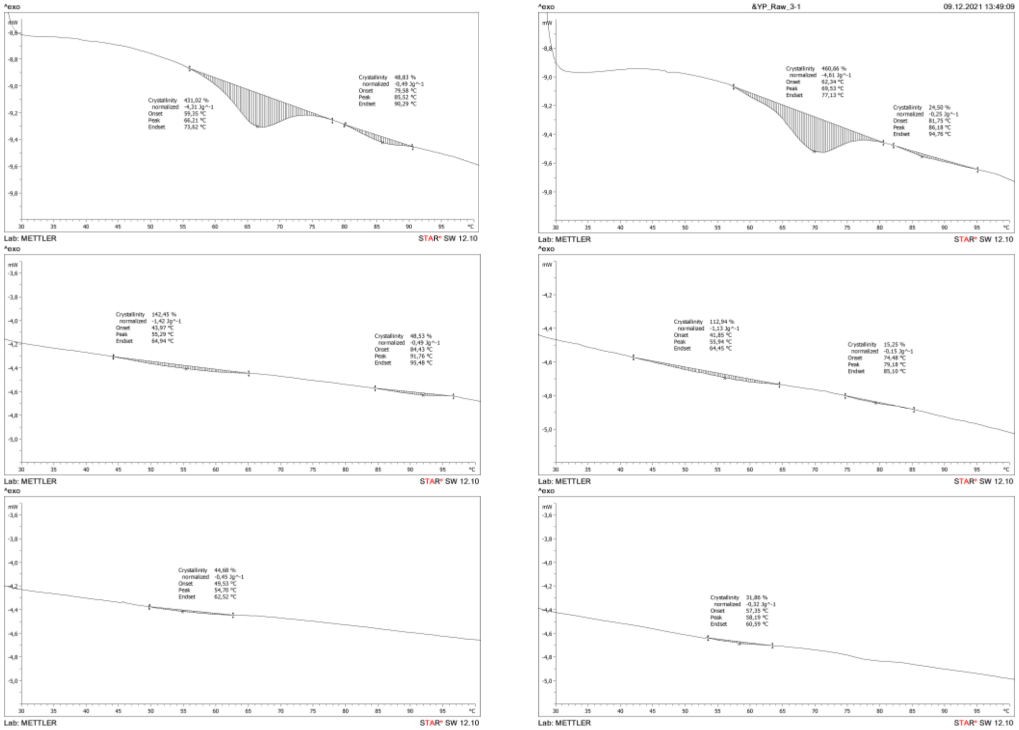

Figure S2. DSC thermograms of red lentil (RL) and yellow pea (YP) flours, second-generation and third-generation red lentil and yellow pea products with feed moisture content of 0.20 kg water per kg dry flour (MC0.20) at 100 °C die temperature (DT100).

Table S1. Pearson correlation coefficients between extrusion variables and extrudate properties in red lentil (RL) third-generation products.

	MC	Torque	Die pressure	SME	EI	Density	L*	a*	b*	ΔE	Hardness	Crunchiness	Crispiness	T_0	T_p	ΔH
DT	0.000	-0.446	-0.832	-0.893	0.700	-0.954	-0.781	0.903	0.683	-0.757	0.213	0.470	0.358	0.685	0.806	-0.620
MC		-0.893	-0.532	-0.446	0.254	-0.129	0.417	-0.322	-0.522	0.373	0.019	0.266	0.150	0.677	0.281	-0.688
Torque			0.852	0.801	-0.424	0.367	0.002	-0.115	-0.180	-0.061	0.426	-0.350	0.093	-0.903	-0.594	0.912
Die pressure				0.986	-0.749	0.726	0.514	-0.608	-0.639	0.442	0.268	-0.490	-0.034	-0.905	-0.777	0.910
SME					-0.757	0.812	0.578	-0.669	-0.679	0.513	0.204	-0.482	-0.031	-0.906	-0.828	0.881
EI						-0.825	-0.420	0.587	0.453	-0.393	0.144	0.526	0.401	0.674	0.758	-0.459
Density							0.688	-0.813	-0.583	0.671	-0.181	-0.499	-0.322	-0.694	-0.871	0.488
L*								-0.961	-0.746	0.992	0.134	0.138	0.240	-0.219	-0.453	0.296
a*									0.791	-0.939	-0.104	0.003	-0.117	0.330	0.536	-0.392
b*										-0.656	-0.320	-0.018	-0.216	0.423	0.668	-0.466
ΔE											0.091	0.153	0.232	-0.136	-0.355	0.218
Hardness												0.254	0.757	-0.214	0.038	0.529
Crunchiness													0.689	0.565	0.777	-0.310
Crispiness														0.110	0.383	0.195
T_0															0.828	-0.857
T_p																-0.627

DT: Die temperature; MC: Feed moisture content; SME: Specific mechanical energy; EI: Expansion index; L*: Darkness/lightness; a*: Greenness/redness; b*: Blueness/yellowness; ΔE: Overall colour difference; T_0: Onset temperature; T_p: Peak temperature; ΔH: Enthalpy of starch gelatinization.

Table S2. Pearson correlation coefficients between extrusion variables and extrudate properties in yellow pea (YP) third-generation products.

	MC	Torque	Die pressure	SME	EI	Density	L*	a*	b*	ΔE	Hardness	Crunchiness	Crispiness	T_0	T_p	ΔH
DT	0.000	-0.473	-0.531	-0.850	0.431	-0.559	-0.195	0.696	0.392	-0.090	-0.328	0.226	-0.112	-0.092	0.100	-0.572
MC		-0.850	-0.819	-0.473	-0.231	0.665	-0.691	0.483	0.642	-0.691	0.211	-0.044	0.136	0.204	-0.286	-0.726
Torque			0.988	0.821	0.164	-0.395	0.788	-0.550	-0.577	0.882	-0.472	-0.607	-0.651	-0.031	0.205	0.879
Die pressure				0.849	0.163	-0.377	0.834	-0.600	-0.638	0.917	-0.414	-0.584	-0.642	-0.057	0.084	0.886
SME					-0.023	0.094	0.964	-0.864	-0.895	0.963	-0.011	-0.152	-0.260	0.017	0.163	0.826
EI						-0.605	0.213	0.168	-0.076	0.276	-0.137	0.173	-0.018	-0.278	0.105	0.161
Density							-0.369	-0.208	0.180	-0.453	0.285	-0.129	0.224	0.045	0.018	-0.262
L*								-0.741	-0.951	0.989	-0.017	-0.156	-0.331	-0.023	-0.014	0.827
a*									0.878	-0.645	-0.081	0.254	0.299	-0.018	0.067	-0.611
b*										-0.893	-0.043	0.179	0.322	-0.061	0.097	-0.690
ΔE											-0.048	-0.137	-0.324	-0.071	0.036	0.875
Hardness												0.072	0.727	-0.263	0.114	-0.155
Crunchiness													0.548	0.079	-0.009	-0.464
Crispiness														0.006	0.127	-0.532
T_0															-0.213	-0.245
T_p																0.264

DT: Die temperature; MC: Feed moisture content; SME: Specific mechanical energy; EI: Expansion index; L^*: Darkness/lightness; a^*: Greenness/redness; b^*: Blueness/yellowness; ΔE: Overall colour difference; T_o: Onset temperature; T_p: Peak temperature; ΔH: Enthalpy of starch gelatinization.
